# Non-targeted metabolomic profiling of *Cremastra appendiculata* providing insights for phytochemical analyses

**DOI:** 10.7717/peerj.20592

**Published:** 2026-01-08

**Authors:** Rui Guan, Yuxin Shan, Hamizah Shahirah Hamezah, Somnuk Bunsupa, Hong To Quyen Duong, Yadong Zhou, Rongchun Han, Xiaohui Tong

**Affiliations:** 1 School of Pharmacy, Anhui University of Chinese Medicine, Hefei, China; 2 Institute of Systems Biology, Universiti Kebangsaan Malaysia, Bangi, Malaysia; 3 UKM-AHUCM Joint Laboratory for Traditional Medicine Quality Standardization, Universiti Kebangsaan Malaysia, Bangi, Malaysia; 4 Faculty of Pharmacy, Mahidol University, Bangkok, Thailand; 5 Scientific Research—Healthcare Activity Direction Department, Traditional Medicine Hospital of Ho Chi Minh City, Xuan Hoa Ward, Vietnam; 6 School of Humanities and International Education & Exchange, Anhui University of Chinese Medicine, Hefei, China; 7 AHUCM-UKM Joint Laboratory for Traditional Medicine Quality Standardization, Anhui University of Chinese Medicine, Hefei, China; 8 School of Life Sciences, Anhui University of Chinese Medicine, Hefei, China; 9 Functional Activity and Resource Utilization on Edible and Medicinal Fungi Joint Laboratory of Anhui Province, Jinzhai, China

**Keywords:** *Cremastra appendiculata*, Untargeted metabolomics, Quality markers, Tissue-specific profiling

## Abstract

**Background:**

*Cremastra appendiculata* (D. Don) Makino, known as “Shan Cigu” in China, is a valuable medicinal plant historically employed for its antibacterial and anti-inflammatory properties. However, its comprehensive metabolome remains underexplored, hindering the establishment of standardized quality control.

**Methods:**

In this study, a non-targeted metabolomics approach based on the Thermo Fisher Orbitrap Exploris 120 LC-MS (Liquid Chromatography-Mass Spectrometry) platform was employed to systematically profile the metabolites of * C. appendiculata*.

**Results:**

A total of 174 compounds were annotated through a dual-validation workflow integrating Compound Discoverer 3.3 and manual tandem mass spectrometry spectral verification. Orthogonal partial least squares discriminant analysis prioritized 30 candidate quality markers, of which, six were further validated through network pharmacology-based bioactivity screening. Hierarchical clustering analysis revealed distinct metabolic patterns across the different tissues (roots, pseudobulbs, and leaves), establishing a tissue-specific chemical atlas. The integration of chemometric, network pharmacological, and chemotaxonomic analyses resulted in a robust, molecularly guided quality control framework, providing novel insights for phytochemical research and medical applications of *C. appendiculata*.

## Introduction

*Cremastra appendiculata* (D. Don) Makino, a perennial herbaceous plant of the Orchidaceae family, has been historically utilized in China as the primary botanical source for the traditional Chinese medicine Cremastrae Pseudobulbus Pleiones Pseudobulbus (CPPP), known in Chinese as “Shan Cigu”. According to traditional Chinese medicine theory, the pseudobulb of *C. appendiculata* undergoes specific processing to produce CPPP for clinical applications (Jiang et al., 2022). The medicinal properties of *C. appendiculata* were documented in The Supplement to the Compendium of Materia Medica (1765) by Xuemin Zhao, who emphasized its antimicrobial, anti-inflammatory, antioxidant, and immunomodulatory effects. In modern clinical practice, it is used to treat tuberculous lymphadenitis, refractory abscesses, gouty arthritis, early-stage breast hyperplasia, and other conditions (Norimoto et al., 2021). Recent studies have also reported its anticancer effects against breast cancer and other malignancies (Cao et al., 2021; Liu et al., 2021; Wu, Yan & Kang, 2024). For example, compounds isolated from *C. appendiculata*, such as gastrodin and batatasin III, have been shown to induce protective autophagy and apoptosis in cancer cells *via* specific signaling pathways (Cao et al., 2024; Pinkhien et al., 2017; Qin et al., 2021).

As a member of Orchidaceae, a plant family renowned for its ecological and medicinal value, *C. appendiculata* shares research interest with other orchids owing to its abundant secondary metabolites (*e.g.*, polysaccharides, phenanthrenes, and bibenzyls). These metabolites form the foundation of natural drug development and have attracted sustained research attention in China. For instance, similarities in polysaccharide composition between *Dendrobium huoshanense* and *C. appendiculata* suggest potential cross-species insights (Han et al., 2018)*.* Current research on *C. appendiculata* primarily focuses on three aspects: (1) symbiotic interactions with mycorrhizal fungi, which are essential for the propagation and conservation of its wild resources; (2) the development of rapid artificial cultivation techniques aimed at meeting market demand and alleviating pressure on wild populations; and (3) the quantification, distribution patterns, and accumulation dynamics of specific bioactive compounds such as phenanthrenes, including representative compounds like flavanthrinin, which provide a scientific basis for elucidating its medicinal value (Pan et al., 2024; Xue et al., 2006; Yang et al., 2022). However, the holistic metabolome of the entire plant remains underexplored, with numerous bioactive constituents yet to be fully characterized. Furthermore, the lack of standardized quality control criteria and molecular-level quality assessment frameworks hinders the establishment of precise phytochemical evaluation systems.

Metabolomics, a high-throughput systems biology approach integrating hyphenated analytical platforms and multivariate statistical models, enables comprehensive characterization of low-molecular-weight metabolites (<1,500 Da) to map dynamic metabolic networks shaped by genetic–environmental interactions. In plant chemical biology, this approach has revolutionized phytochemical investigations by resolving species-specific metabolic signatures and pharmacologically active constituents. Systematic profiling of bioactive compounds such as alkaloids, terpenoids, and phenolics, not only identifies the molecular determinants underlying plant-derived therapeutic efficacy, but also establishes a holistic chemical blueprint essential for validating ethnopharmacological claims. Non-targeted metabolomics further advances plant research *via* tissue-specific metabolic cartography, generating chemical atlases that reveal authenticated biomarkers and structurally novel compounds with unknown bioactivity. Importantly, metabolomics transcends conventional phytochemical analyses by establishing a molecular-guided ethnopharmacological framework that (1) correlates metabolic fingerprints with historical therapeutic applications to identify bioactive efficacy markers, (2) guides agronomic optimization through developmental stage-resolved metabolite tracking, and (3) forms conservation genomics by discriminating between wild and cultivated chemotypes. Accordingly, the application of non-targeted metabolomics can enhance the identification of *C. appendiculata* metabolites, facilitate the screening of quality markers (Q-markers) through bioactivity-guided prioritization and multi-omics validation, and integrate and visualize the study content, thus establishing a molecularly defined quality-control framework (Gertsman & Barshop, 2018; Hong et al., 2016; Kumar, Bohra & Kumar, 2017).

Liquid chromatography-mass spectrometry (LC-MS), with its high sensitivity and resolution, enables precise profiling of bioactive components, aiding Q-marker identification, pharmacological mechanism elucidation, and sustainable resource utilization. The Thermo Fisher Orbitrap Exploris 120 mass spectrometer used in this study offers ultrahigh resolution, high sensitivity with a broad dynamic range, rapid scanning speed, multimode compatibility, and multistage fragmentation. Compared with quadrupole time-of-flight mass spectrometry (Q-TOF MS) and triple quadrupole mass spectrometry (TQMS), its ultra-high resolution (120,000 full width at half maximum, FWHM) and sub-ppm mass accuracy enable the precise differentiation of structurally similar compounds, such as various flavonoids with a similar parent nuclear structure. Its broad dynamic range (>5 orders of magnitude) and femtogram (fg)-level sensitivity allow comprehensive analysis spanning from polysaccharides to trace alkaloids, outperforming the detection limits of ion trap instruments. Furthermore, rapid scanning (15 Hz) and compatibility with multistage fragmentation make it ideal for non-targeted metabolomic studies, providing robust data for Q-marker screening (Cooper & Yang, 2024).

By integrating metabolomics and LC-MS, this study systematically characterized the complex metabolome of *C. appendiculata* and identified 174 annotated compounds. Orthogonal partial least squares discriminant analysis (OPLS-DA) prioritized 30 candidate Q-markers, with six validated markers selected through network pharmacology-based target pathway verification. Multivariate analyses (principal component analysis (PCA) and hierarchical clustering analysis (HCA) revealed tissue-specific metabolic clusters between pseudobulbs and non-medicinal organs, establishing a molecular-guided quality control framework for the sustainable utilization and conservation of this endangered Orchidaceae species (Li et al., 2016; Pezzatti et al., 2020).

## Materials & Methods

### Plant materials and reagents

Wild *C. appendiculata* specimens (six plants) were collected in Yuexi County, Anqing City, Anhui Province, China, in August 2024. The collected materials were immediately flash-frozen in liquid nitrogen and transported to the laboratory on dry ice. After taxonomic cleaning, roots, pseudobulbs, and leaves were separated (six replicates each), labeled as R1-6 (roots), P1-6 (pseudobulbs), and L1-6 (leaves), respectively, and stored at −80 °C until use. In this study, six standard chemicals were adopted for identification of interested components (Supplementary material 1). LC-MS grade methanol and formic acid (Merck, Rahway, NJ, USA) were used for mobile phase preparation. Ultra-pure water was produced using a PALL Cascada lab water purification system (PALL, Port Washington, NY, USA).

### Instrumentation and chromatographic-mass spectrometry conditions

#### Liquid chromatography system

A Vanquish UPLC system (Thermo Fisher Scientific, Waltham, MA, USA) equipped with an ACQUITY UPLC HSS T3 column (2.1 mm × 100 mm, 1.8 µm; Waters, Milford, MA, USA) was employed. The mobile phase comprised (A) water containing 0.1% formic acid and (B) pure methanol. The gradient elution program was as follows: 0–3 min, 3% B; 3–8 min, 3–45% B; 8–18 min, 45–65% B; 18–20 min, 65–90% B; 20–24 min, 90–100% B; 24–25 min, 100–3% B; 25–30 min, 3% B. The flow rate was 0.3 mL/min, the column temperature was maintained at 30 °C, the autosampler temperature was set to 10 °C, and the injection volume was four µL.

#### High-resolution mass spectrometry system

Mass spectrometry (MS) was performed using an Orbitrap Exploris 120 system with an electrospray ionization (ESI) source. ESI parameters were as follows: spray voltage, ±3,000 V (positive/negative ion mode switching); sheath gas N_2_ flow rate, 40 Arb; auxiliary gas, 7 Arb; purge gas, 1 Arb; ion transfer tube temperature, 320 °C; vaporizer temperature, 325 °C. Full-scan mass range was set to m/z 100–1500 with a resolution of 60,000 (at m/z 200) and a signal intensity threshold of 2 ×10^6^. Data-dependent acquisition (DDA) triggered tandem mass spectrometry (MS/MS) scans were performed with the following settings: precursor isolation window, two m/z; HCD collision energy, three levels 20, 40, and 60 eV; MS/MS resolution, 15,000.

### Sample preparation

Root, pseudobulb, and leaf tissues (50 mg each) were placed into pre-chilled 1.5 mL *Eppendorf* tubes and homogenized using a ball mill. Subsequently, one mL of 80% methanol was added, followed by vortex mixing and ultrasonication in a water bath at 40 °C for 30 min to facilitate extraction. The mixture was centrifuged (12,000 rpm, 20 °C, 10 min) and the supernatant was collected. This process was repeated until no precipitate was observed, and the supernatant was subsequently stored in a refrigerator at −20 °C until further analysis. A standard stock solution (50 µg/mL) was prepared in 80% methanol and processed following the same protocol. To evaluate system stability, 50 µL aliquots from 18 samples (R1-6, P1-6, L1-6) were pooled and equally divided into three quality control (QC) solutions. One QC sample was injected after the elution of every six experimental samples to monitor the retention time and peak intensity deviations (RSD <15%).

### Data processing and statistical analysis

A systematic workflow was employed for data processing and statistical analysis to enable in-depth mining of mass spectrometry data from *C. appendiculata*. Raw data were acquired using Xcalibur 4.1.31 software and subsequently subjected to multidimensional analysis in three progressive stages. First, a consolidated annotation strategy was applied through the Compound Discoverer 3.3 platform, which combined in-house databases, improving the annotation of prioritized compounds, with public repositories (mzCloud and ChemSpider) to broaden compound coverage. Data from both positive and negative ion modes were processed separately and then merged, with duplicates removed based on the retention time and fragmentation patterns. This process resulted in the identification of 174 representative compounds.

In the analytical setup, peak selection was primarily based on mass spectrometry features (mass-to-charge ratio (m/z), retention time (RT)) and signal characteristics. Raw data underwent preprocessing through baseline correction and noise filtering, followed by algorithmic detection of qualified signal peaks in consecutive scans (with thresholds set at S/N ≥ 5, minimum peak intensity of 10^4^, and a peak width range of 0.2 min). Isotopic peaks were grouped based on isotopic distribution models to consolidate signals from the same compound, and retention time alignment was applied to correct for minor RT variations across samples for consistent peak matching. High mass accuracy was utilized to further reduce isobaric interference, ultimately yielding reliable peaks of biological and chemical relevance. For missing values, features with excessive gaps were filtered using a predefined threshold. The core approach combines K-nearest neighbors (KNN, *K* = 5) and retention time-correlated imputation (RT tolerance = 0.1 min), supplemented by strategies incorporating background noise and isotopic distribution to handle low-abundance missing values. All imputed values were annotated to ensure traceability. The overall workflow was tailored to the characteristics of MS data, effectively balancing processing efficiency and accuracy.

A two-tiered screening model was subsequently established. Initial screening *via* partial least squares discriminant analysis (PLS-DA) in Compound Discoverer 3.3 identified 30 candidate biomarkers with intergroup differentiation potential. Orthogonal Partial Least Squares Discriminant Analysis (OPLS-DA) validation was subsequently performed using SIMCA 14.1. Fourteen candidate Q-markers were finally selected based on VIP > 1.0, *p* < 0.05, structural uniqueness, and biological activity. The highest-ranking compounds were identified using Technique for Order Preference by Similarity to an Ideal Solution (TOPSIS) analysis. Model validation included 200 permutation tests (*p* < 0.01) to assess significance and prevent overfitting (R^2^Y > 0.8 indicating model explanatory power; Q^2^ > 0.5 indicating predictive capability). Visualization analysis performed in Origin 2021 included HCA, revealing metabolite abundance gradients across roots, leaf, and pseudobulb tissues. Heat maps were generated using TBtools (version 2.0) to illustrate metabolite distribution patterns. Chemotaxonomic classification grouped compounds into flavonoids, glycosides, and phenolic acids and others, with molecular structures drawn in ChemDraw 19.0 and integrated into composite visualizations, thereby enabling an intuitive overview of the *C. appendiculata* metabolome.

## Results

### High-confidence metabolite annotation *via* dual validation

Comprehensive metabolite profiling of *C. appendiculata* was performed using an Orbitrap Exploris 120 LC–MS system in data-dependent acquisition (DDA) mode, providing high-resolution mass accuracy (mass error <2 ppm) and MS/MS fragmentation data. Raw data were processed with Compound Discoverer 3.3, following a structured annotation workflow comprising: (1) peak alignment and deconvolution, (2) molecular formula prediction (m/z tolerance ± 3 ppm), (3) database matching against mzCloud and ChemSpider, and (4) retention time prediction based on polarity-specific elution behavior. Following integrative screening of in-house and public databases, 174 metabolites were identified. To ensure high reliability, all identifications underwent a dual validation process. Automated scoring in Compound Discoverer (match score >70%) was supplemented by manual verification of MS/MS spectra against literature data or authentic standards. For instance, the identification of rutin was confirmed in negative ion mode by its diagnostic fragment ions. This stringent validation yielded a refined list of 174 high-confidence metabolites including flavonoids, phenolic acids, and lipid derivatives. Detailed information on these compounds is provided in Supplementary Materials 2 and 3.

**Figure 1:**
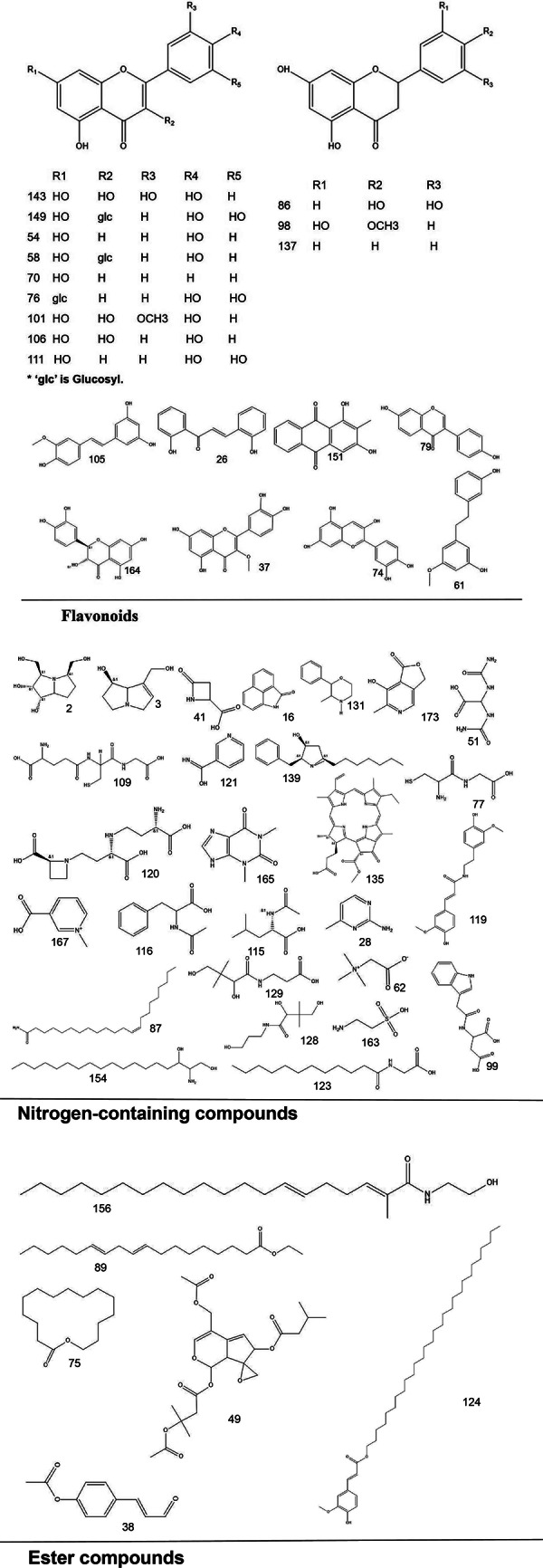
The structural formula diagrams of flavonoid compounds, nitrogen-containing compounds, and ester compounds among the identified compounds.

**Figure 2:**
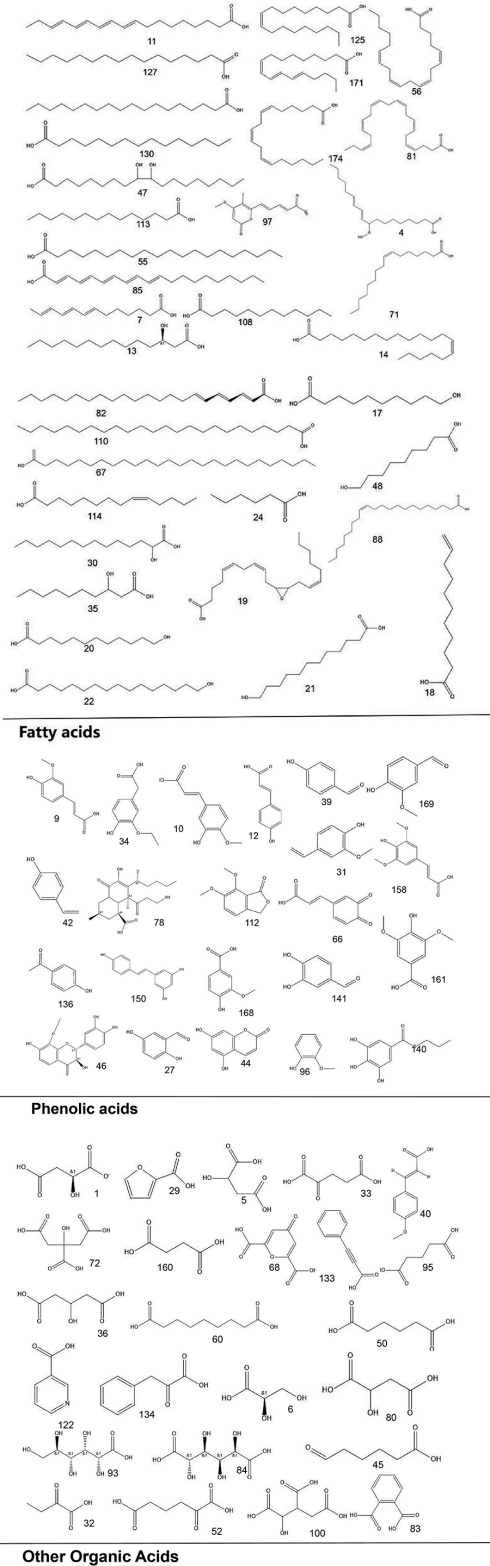
The structural formula diagrams of fatty acid compounds, phenolic acid compounds, and other organic acid compounds among the identified compounds.

**Figure 3:**
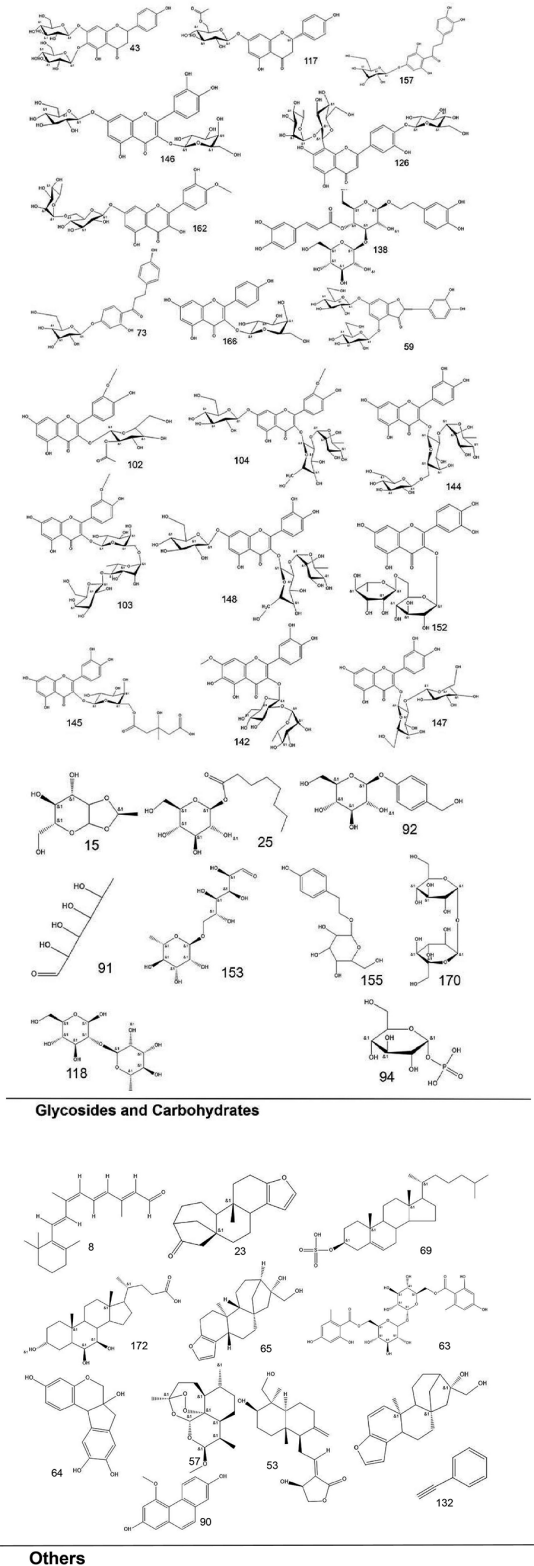
The structural formula diagrams of glycoside and carbohydrate compounds, and other compounds among the identified compounds.

To systematically interpret the chemical diversity of *C. appendiculata*, the annotated compounds were classified into eight major groups based on their biosynthetic origins and structural features: flavonoids, glycosides, carbohydrates, fatty acids, phenolic acids, other organic acids, nitrogen-containing compounds, and esters. Structural elucidation was further enhanced by generating molecular scaffolds and core structures using ChemDraw 19.0. For example, within the flavonoid class, numerous quercetin derivatives and analogs were systematically categorized based on their shared 2-phenylchromone backbone. To elucidate structural relationships, we established a standardized nomenclature protocol: (1) Variable substituents in the parent nucleus are assigned position-specific codes (R_1_, R_2_, *etc.*); (2) corresponding compound serial numbers and substituents are listed below the core structure. For compounds that are not suitable for this classification (*e.g.*, compounds that do not have a unified parent nuclear structure or a special chemical formula structure), structural formula diagrams are drawn separately and organized together according to the structural taxonomy. This structural classification clarified metabolic relationships and provided a visual foundation for prioritizing pharmacologically relevant compound clusters in subsequent analyses. Representative structural diagrams of these compounds are shown in Figs. 1–3.

### Tiered screening of quality markers integrating chemometrics and pharmacology

**Figure 4:**
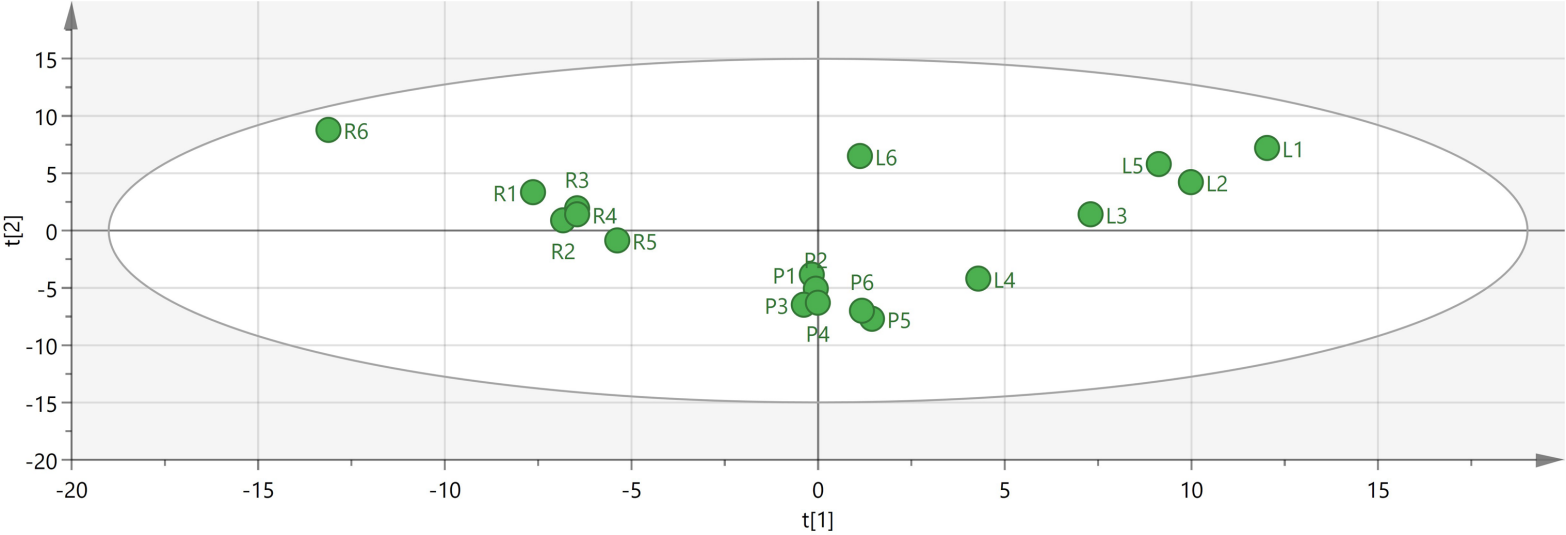
Visual analysis of major components in different tissues of *C. appendiculata*.

**Figure 5:**
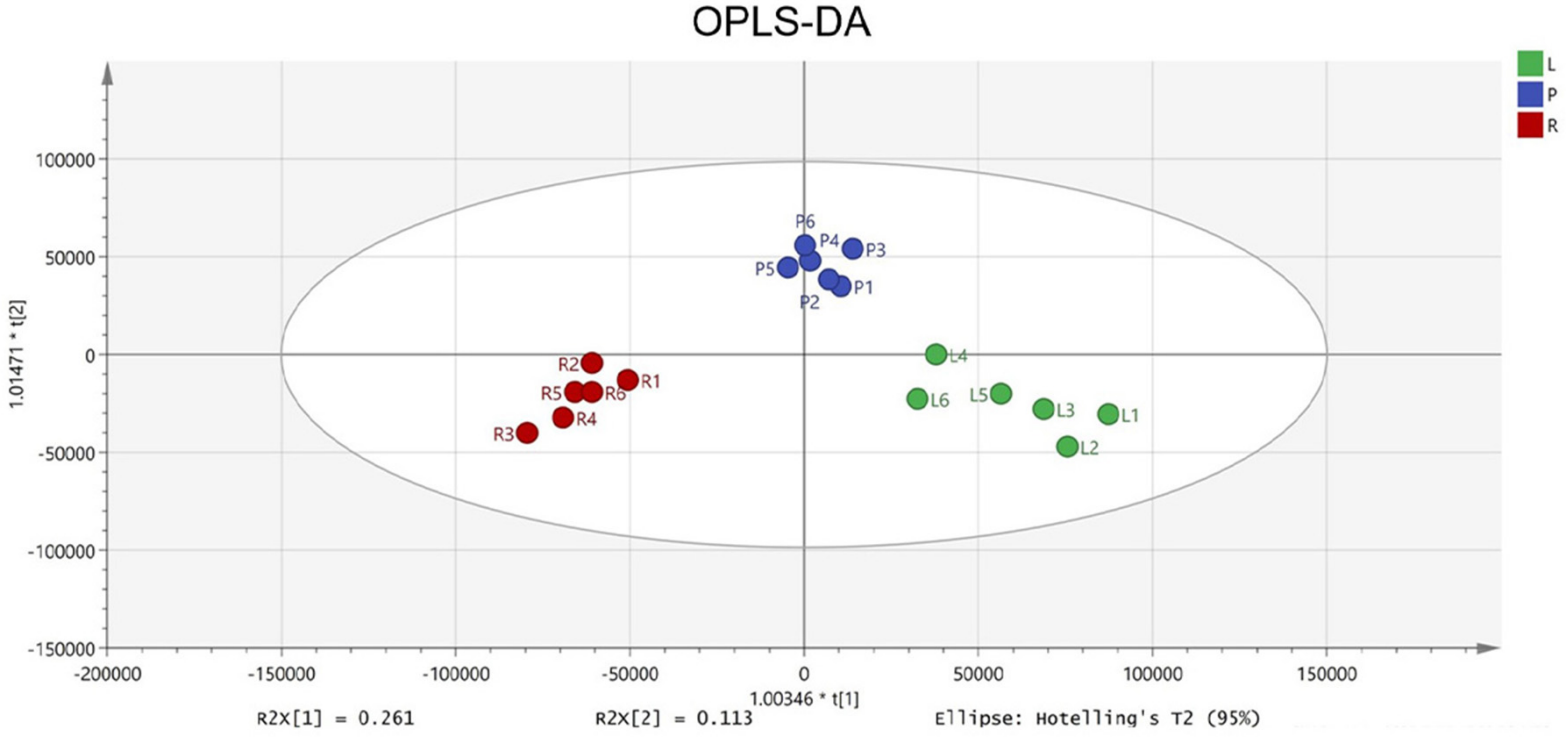
The OPLS-DA score plot indicating significant differences concerning *C. appendiculata* metabolic profile between distinct groups.

**Figure 6:**
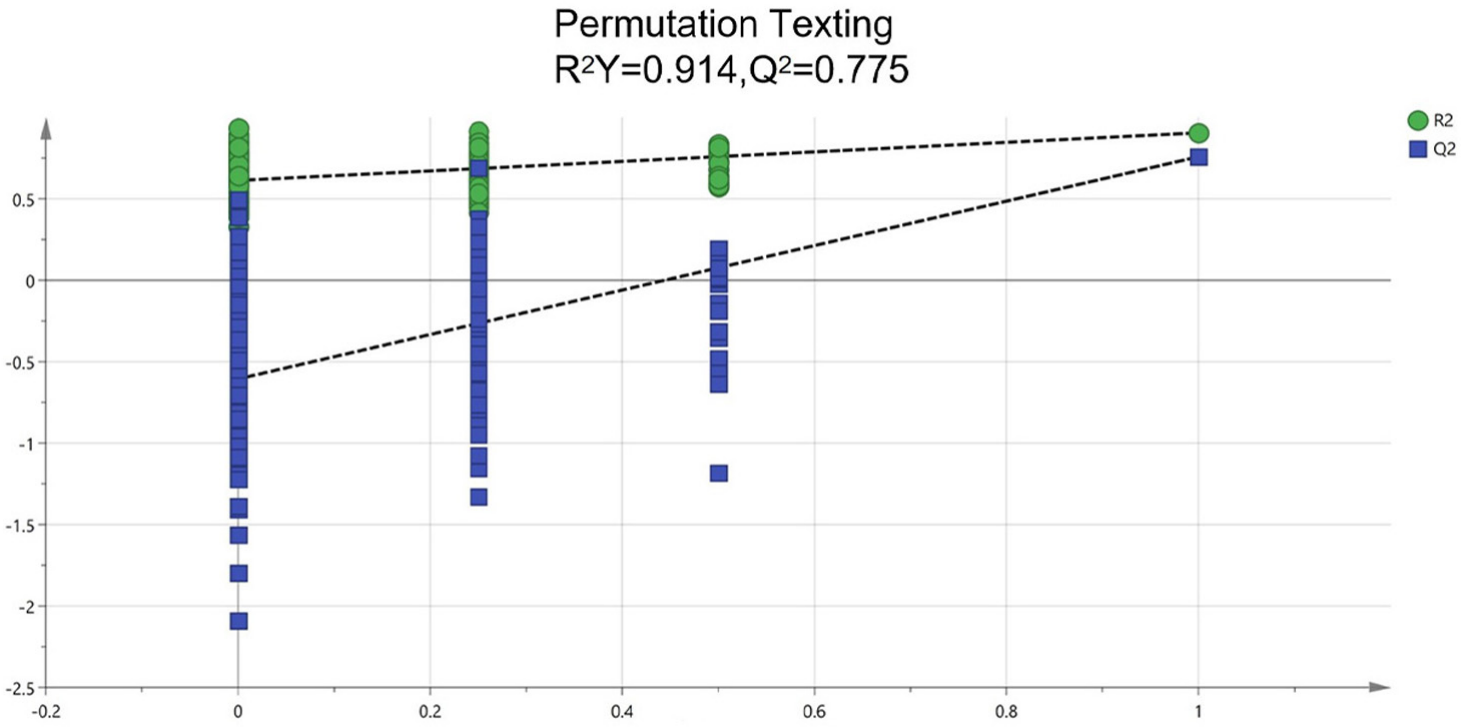
Assessment of reliability for the current OPLS-DA model adopting permutation plot.

To identify potential quality markers (Q-markers), the validated metabolite dataset was first subjected to principal component analysis (PCA), revealing intrinsic metabolic differences among the roots, pseudobulbs, and leaves, with clear clustering by tissue type (Fig. 4). Subsequently, OPLS-DA was employed to maximize the separation between groups and identify the most discriminatory variables. The OPLS-DA model exhibited clear segregation of the three tissue types (Fig. 5) and demonstrated robust predictive capability (R2X = 0.641, R2Y = 0.914, Q2 = 0.775) with significant permutation validation (*p* < 0.001), confirming model robustness and absence of overfitting (Fig. 6).

Thirty candidate biomarkers with VIP values >1.0 were initially extracted. This candidate pool was refined *via* a tiered screening process. First, based on statistical significance (*p* < 0.05) and documented pharmacological relevance (PubMed and TCMSP), the list was narrowed down to 14 candidates. Next, a TOPSIS analysis ranked these 14 compounds using a comprehensive evaluation of their VIP scores, *p*-values, and reported bioactivities. The multi-criteria decision-making process ultimately identified six compounds as definitive Q-markers: 4-Methoxycinnamic acid, cafestol, rutin, tamarixetin 7-rutinoside, trigonelline, and α-eleostearic acid. These six markers were selected not only because of their strong contribution to inter-group discrimination, but also because of their representative bioactivities and structural diversity. For instance, cafestol, 4-Methoxycinnamic acid, and rutin all exhibit anti-inflammatory and antineoplastic properties while representing distinct chemical classes. Cafestol demonstrates antidiabetic effects in murine models and modulates serum cholesterol levels (Mellbye et al., 2017); 4-Methoxycinnamic acid is a widely occurring natural phenolic acid with multiple proven effects, including antibacterial, antifungal, anti-inflammatory, and cancer-inhibiting activities (Wang et al., 2023); rutin exerts neuroprotective effects, particularly in experimental paradigms of Alzheimer’s disease and epilepsy (Al-Dhabi et al., 2015). The rigorous choice of these markers ensures both specificity and comprehensiveness in quality assessment.

**Figure 7:**
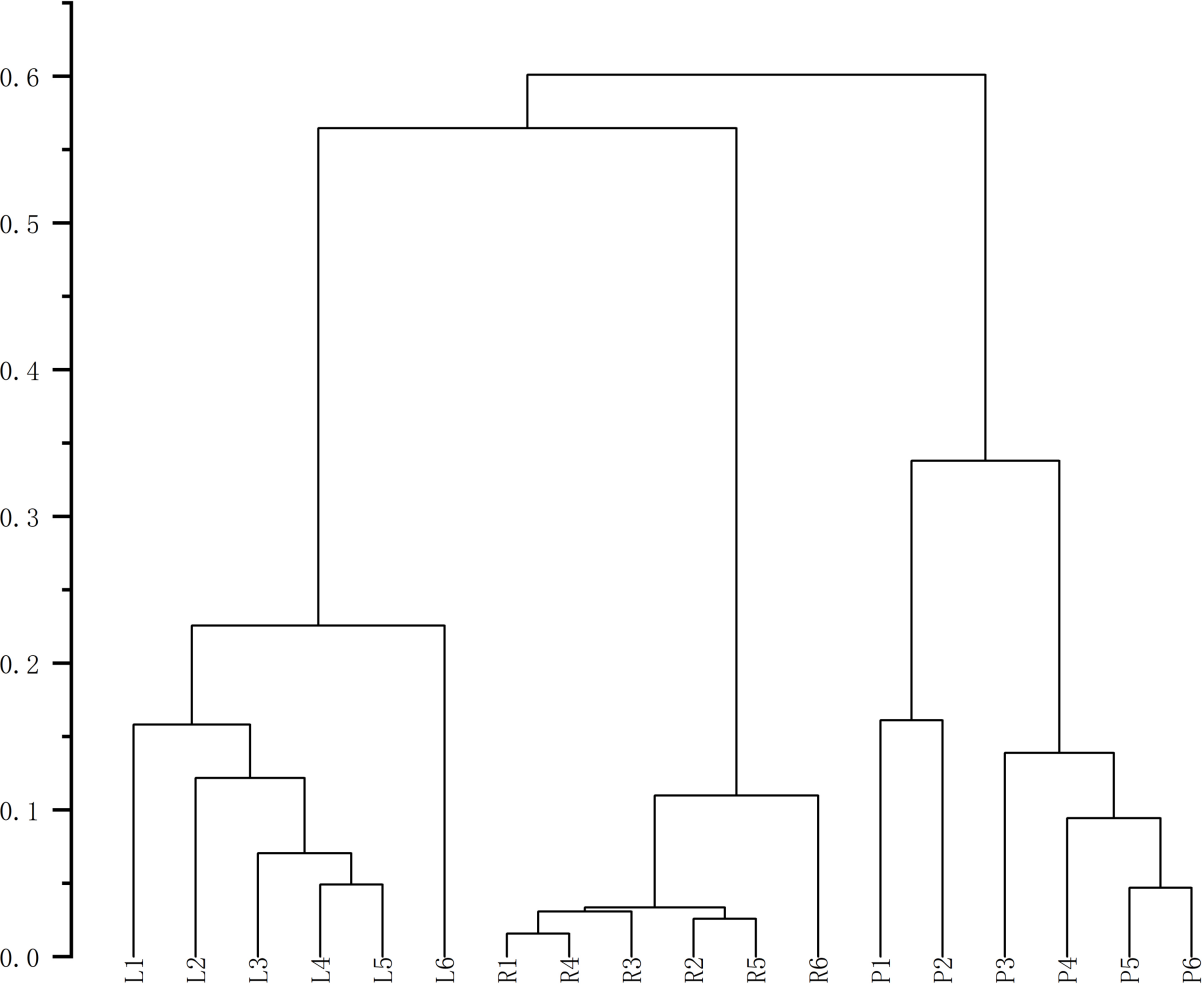
Cluster analysis diagrams generated using Origin 2021.

**Figure 8:**
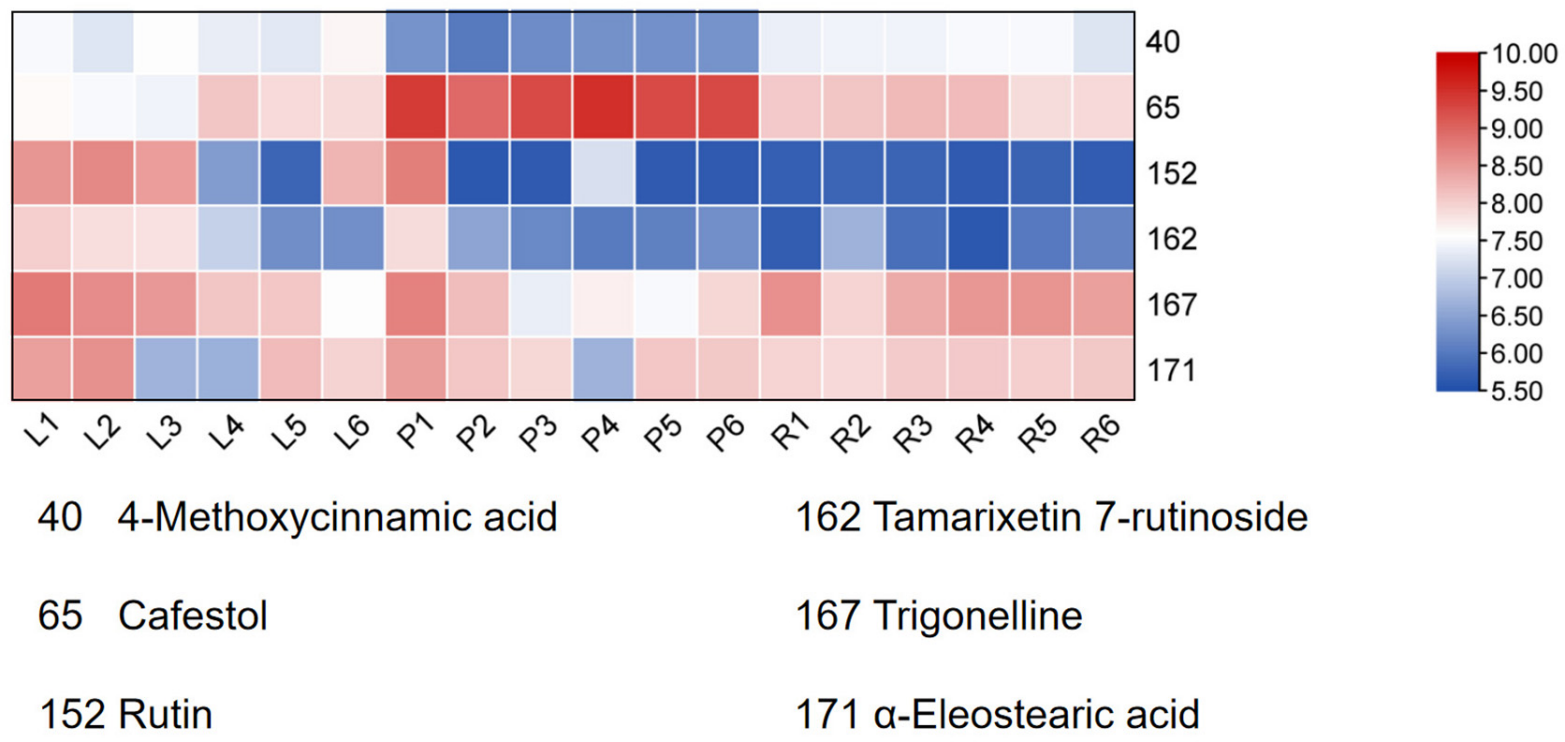
Heatmap of the six Q-markers to visualize their performance in respective tissues. The color bar on the right functions as a log_10_-transformed content scale. The intensity of the red coloration is directly proportional to the substance’s content in the corresponding sample, thereby providing an intuitive comparison of each Q-marker’s performance in the pseudobulb, root, and leaf tissues.

Finally, HCA of the 174 quantified metabolites was performed in Origin (version 2021). Signal intensities were log-transformed and mean-centered for normalization, and clustering was performed using Ward’s linkage method with Euclidean distance to visualize intercomponent relationships (Fig. 7). Heat maps of the six mass markers were generated using TBtools 2.0 (Fig. 8), illustrating their relative distributions and sample marker associations. Collectively, these analyses revealed the underlying biological trends and group-specific metabolic signatures while simultaneously confirming the consistency and robustness of our analytical workflow.

## Discussion

The comprehensive non-targeted metabolomic profiling conducted in this study elucidated the chemical diversity of *C. appendiculata*, overcoming previous limitations in its phytochemical characterization. Leveraging the ultra-high-resolution capabilities of the Orbitrap Exploris 120 platform, 174 metabolites spanning multiple classes—including flavonoids, phenolic acids, and fatty acids—were confidently annotated, thereby enriching the current phytochemical database for *C. appendiculata*.

**Figure 9:**
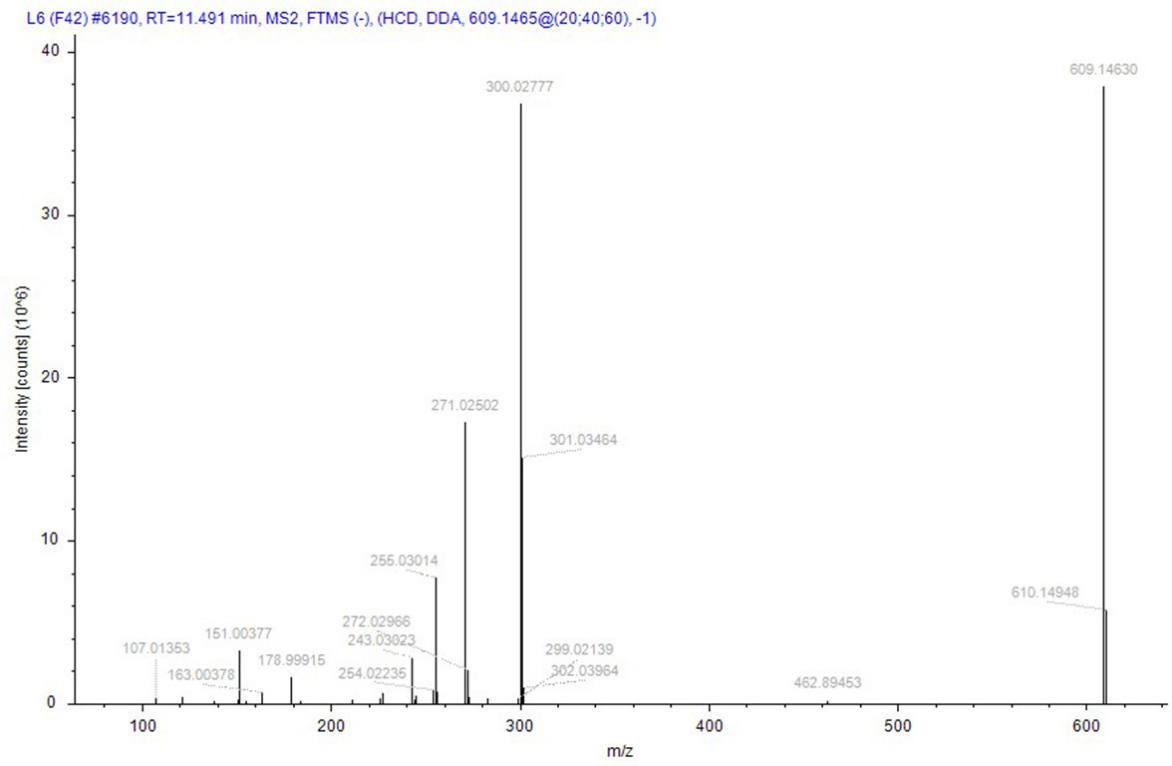
The secondary mass spectrum of rutin serves as evidence for the identification of the compound.

Compound identification followed a rigorous, logically structured validation process. Taking compound 152 (rutin), designated as a quality marker in this study, as an example, its identification was supported by the following evidence: its (M–H)^−^ ion was detected at m/z 609.14660 (theoretical mass for C_27_H_29_O_16_: 609.14556), corresponding to a minimal mass error of +0.81 ppm. The MS/MS spectrum was subsequently matched against the public database mzCloud, which exhibited high similarity (score 87.9) to the reference spectrum of rutin, with a score > 85, indicating high-confidence identification. In addition to automated software matching, manual interpretation of the MS/MS spectrum was performed to verify the identity of rutin. As shown in Fig. 9, the fragmentation behavior aligns with established patterns for flavonoids: the precursor ion (M–H)^−^ (m/z 609) initially underwent cleavage of the glycosidic bond, leading to the loss of a rutinose moiety (or sequential loss of monosaccharides) to yield the aglycone ion (quercetin–H)^−^ at m/z 301. This aglycone then underwent further fragmentation *via* neutral losses (*e.g.*, CO, C_2_H_2_O, and O) or ring cleavages, generating characteristic fragment ions such as m/z 273, 259, and 285. In addition, independent cleavages within the A- and B-rings produced signature fragments with m/z values of 151 and 179, respectively. All the major fragment ions corresponded closely to the expected fragmentation pathway of rutin. Therefore, the MS/MS spectrum was conclusively assigned to rutin, providing high-confidence validation of its identification.

A robust and systematic strategy was developed for screening Q-markers integrating four criteria: the VIP score (derived from OPLS-DA), *p*-value, structural uniqueness, and traditional bioactivity, effectively overcoming the limitations of single-indicator approaches. This synergistic framework delivers distinct core values: differential correlation *via* VIP scores identifies components driving quality disparities, statistical reliability through *p*-values excludes fortuitous differences and reduces false positives, chemical specificity ensured by structural uniqueness avoids cross-species overlap, and functional relevance links chemical markers to historical medicinal use and theoretical efficacy in traditional Chinese medicine. By implementing multiple screening thresholds, this method significantly minimizes both false positives (*e.g.*, components lacking statistical significance or bioactivity) and false negatives (*e.g.*, non-specific or pharmacologically irrelevant compounds), thereby substantially improving screening accuracy (Li et al., 2021; Liu et al., 2023; Yan et al., 2025).

This multidimensional approach bridges modern analytical rigor, combining multivariate statistics and chemoinformatics, with traditional medicinal knowledge, being particularly suitable for complex systems, such as Chinese herbal medicine and ethnopharmacology. Furthermore, integration of VIP scores and *p*-values in metabolomic analysis enables effective discrimination between authentic and adulterated materials; molecular fingerprinting of structurally unique compounds prevents marker overlap with closely related species, and bioactivity correlation ensures pharmacological relevance, collectively reinforcing the practical applicability and scientific robustness of the proposed system (Ren et al., 2020).

While the screening based on the aforementioned four indicators offers several advantages, it also entails a degree of inherent subjectivity. To address this limitation, TOPSIS was introduced to assign weighted composite scores, ranking the initially screened candidates by their relative proximity to an ideal solution. Here, the optimal sample is defined as one in which all indicator values reach their respective maxima. Compounds ranked at the top were selected as final quality markers.

**Figure 10:**
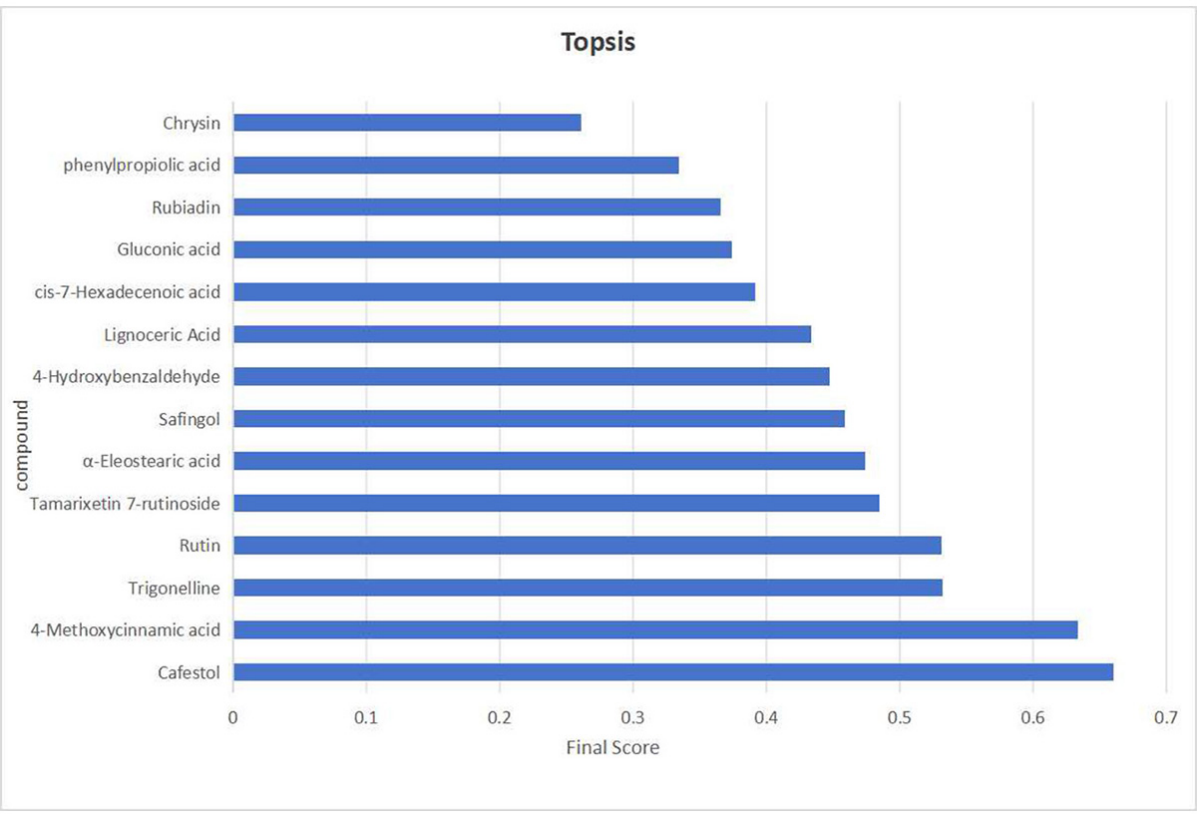
The visualization results of TOPSIS analysis intuitively demonstrate the priority ranking of the 14 compounds.

The initial screening yielded 14 candidate compounds. Their data were processed as follows: *p*-values were transformed to –log_10_ to ensure positivity, while structural uniqueness and activity-related indicators were scaled to a 10-point scoring system. The normalized data were then assigned predetermined weighting coefficients (K): K_VIP_ = K_p_ = 0.35, K_SU_ _(Structural Uniqueness)_ = 0.1, and K_AC_ _(Activity Correlation)_ = 0.2. These values were incorporated into the TOPSIS algorithm to calculate composite scores and generate rankings (Fig. 10). Among the top-ranked compounds, the compound in the seventh place was excluded due to weak association with the traditional efficacy of *C. appendiculata.* Consequently, the top six compounds—4-Methoxycinnamic acid, cafestol, rutin, tamarixetin 7-rutinoside, trigonelline, and α-eleostearic acid—were designated as definitive Q-markers. This approach ensures that the selected markers are not only statistically significant and chemically distinctive, but also pharmacologically relevant, thereby bridging the gap between phytochemical composition and traditional therapeutic efficacy.

The six identified Q-markers, representing diverse biosynthetic pathways (*e.g.*, phenylpropanoids, flavonoids, alkaloids, and fatty acids), collectively enable a holistic quality assessment. Key markers such as rutin, 4-Methoxycinnamic acid, and cafestol have been well documented for their anti-inflammatory and anticancer activities, aligning with the traditional applications of *C. appendiculata*. Their inclusion ensures that the quality control framework is both specific and comprehensive.

While this study establishes a robust foundation for the quality control of *C. appendiculata*, future work should focus on several key areas: (1) expanding the metabolite database to include rare and novel compounds; (2) validating the stability of these Q-markers across different cultivation, harvesting, and processing conditions; and (3) investigating pharmacokinetics and synergistic interactions among the core markers. Such efforts are crucial to translate these chemical insights into precise and reliable quality standards for medicinal plants.

## Conclusion

In this study, a reproducible and systematic framework for Q-marker discovery in *C. appendiculata* was established by integrating non-targeted metabolomics and advanced data analysis strategies. Three key innovations underpin the success of this framework: (1) the ultra-high mass accuracy of the Orbitrap Exploris 120 system minimizes false positive identifications and improves metabolite annotation reliability; (2) the dual-tiered annotation strategy, combining automated Compound Discoverer 3.3 workflows with manual MS/MS spectral validation, enhances the confidence of metabolite identification; and (3) the sequential screening process, integrating chemometric prioritization and network pharmacology-based bioactivity filtering, ensuring both analytical robustness and therapeutic relevance of the selected Q-markers.

Six Q-markers were identified, providing a scientific basis for the standardization of *C. appendiculata* preparations. Moreover, the identification of compounds, such as rutin, 4-Methoxycinnamic acid, and cafestol, elucidates the polypharmacological mechanisms underlying the traditional therapeutic applications of *C. appendiculata*, particularly the synergistic interplay between terpenoid-mediated antitumor activity and flavonoid-driven antioxidant effects.

Future research should focus on expanding the metabolite reference database to encompass rare and previously unreported compounds, validate Q-marker stability under diverse processing and storage conditions, and investigate pharmacokinetic interactions among key markers. These efforts will further refine the molecular quality-control framework and promote the integration of traditional Chinese medicinal knowledge into modern precision medicine paradigms.

##  Supplemental Information

10.7717/peerj.20592/supp-1 Supplemental Information 1 Standard substances applied and their specifications

10.7717/peerj.20592/supp-2 Supplemental Information 2 Mass spectrometry and liquid chromatography information for the 174 compounds identified

10.7717/peerj.20592/supp-3 Supplemental Information 3 Data from the preliminary processing of 174 compounds

## Additional Information and Declarations

### Data Availability

The following information was supplied regarding data availability: The data is available at MetaboLights: MTBLS13252.
